# Diagnosing dementia and cognitive dysfunction in the elderly in primary health care: A systematic review

**DOI:** 10.1590/1980-57642018dn13-020002

**Published:** 2019

**Authors:** Lucas N.C. Pelegrini, Gabriela M.P. Mota, Caio F. Ramos, Edson Jesus, Francisco A.C. Vale

**Affiliations:** 1 PhD student on the Graduate Program in Fundamental Nursing - Nursing School of Ribeirão Preto/ University of São Paulo (EERP/USP), Ribeirão Preto, SP, Brazil.; 2 Master’s student on the Graduate Program in Nursing - Federal University of São Carlos (UFSCar), São Carlos, SP, Brazil.; 3 Physical Educator, Limeira, SP, Brazil.; 4 Professor on the Graduate Program in Nursing - Federal University of São Carlos (UFSCar), São Carlos, SP, Brazil.

**Keywords:** diagnosis, dementia, cognitive dysfunction, primary health care, diagnóstico, demência, disfunção cognitiva, atenção primária à saúde

## Abstract

Dementia is a public health issue making the screening and diagnosing of dementia and its prodromal phases in all health settings imperative.

**Objective:**

using PRISMA, this systematic review aimed to identify how low-, middle-, and high-income countries establish dementia and cognitive dysfunction diagnoses in primary health care.

**Methods:**

studies from the past five years in English, Spanish, and Portuguese were retrieved from Scopus, PubMed, Embase, Lilacs, Scielo, and Web of Science. Of 1987 articles, 33 were selected for analysis.

**Results:**

only three articles were from middle-income countries and there were no studies from low-income countries. The most used instrument was the Mini-Mental State Examination (MMSE). Mild Cognitive Impairment (MCI) and dementia criteria were based on experts’ recommendation as well as on the Diagnostic and Statistical Manual of Mental Disorders (DSM) and International Classification of Diseases (ICD-10), respectively.

**Conclusion:**

differences between these criteria among high- and middle-income countries were observed.

## RESUMO.

Demência é uma questão de saúde pública logo, rastrear e diagnosticar demência e suas fases prodrômicas em todos os níveis de atenção à saúde é imperativo.

**Objetivo:**

uilizando o PRISMA, esta revisão sistemática verificou como os países de baixa, média e alta renda realizam o diagnóstico de demência e disfunção cognitiva na atenção primária.

**Métodos:**

estudos dos últimos cinco anos, em inglês, português e espanhol foram obtidos no Scopus, PubMed, Embase, Lilacs, Scielo, e Web of Science. De 1987 artigos, 33 foram selecionados para a análise.

**Resultados:**

três artigos eram de países de média renda e nenhum de baixa renda. O Mini-Exame do Estado Mental (MEEM) foi o instrumento mais utilizado. Os diagnósticos de Comprometimento Cognitivo Leve (CCL) e demência foram baseados em recomendações de especialistas e no Manual Diagnóstico e Estatístico de Transtornos Mentais (DSM)/Classificação Internacional de Doenças (CID-10), respectivamente.

**Conclusão:**

houve diferenças para estes critérios entre países de alta e média renda.

Dementia is defined by the World Health Organization as a syndrome, usually chronic and progressive, with different causes.^1^ It is a complex condition that affects cognition, behavior, and the autonomy for practicing activities of daily living.^2^ Currently, 50 million people are living with dementia, and projections suggest that this number will triple by 2050, affecting 152 million people.^3^ Alzheimer’s disease (AD) is one of the most common causes of this syndrome.^2^

Cognitive dysfunction, such as mild cognitive impairment (MCI), can be considered a prodromal manifestation of dementia and can be identified years before dementia onset.^4^ The prevalence of MCI in older adults ranges from 15 to 20%, and this condition may be related to high levels of amyloid protein, a biomarker for neurodegeneration and increased risk for dementia.^5^^,^ 6

Little is known about the actual prevalence of dementia.^2^ However, it is known to be more common in women and has a prevalence of 5% in people aged over 65 and up to 32% in elderly aged 85 or older.^1^ In addition, a relationship has been observed between dementia and increased risk for cardiovascular diseases, metabolic syndrome, and neuropsychiatric disorders.^7^^,^ 8

Another intriguing fact about dementia syndromes is underreporting rates, which are higher in low- and middle-income countries (93.2% in Asia, 62.9% in North America, 53.7% in Europe).^9^ Usually, the delay for establishing dementia diagnosis is about 29-37 weeks between symptoms onset and definitive clinical diagnosis.^10^

In this context, primary health care represents the first and closest contact between the elderly and health system, as well as being fundamental for the development of strategies for early identification of diseases.^8^ On the other hand, numerous factors have been suggested as causes for late diagnosis of dementia: normal cognitive changes expected in the aging process, patients’ low educational level, and lack of professional training for correct interpretation of neuropsychiatric symptoms.^8^^,^ 10 ^,^ 11

Given the importance of early diagnosis for dementia and cognitive dysfunction (i.e. MCI), as well as the fact that primary health care settings are the entry point to the health system, the aim of this systematic review was to identify how low-, middle-, and high-income countries establish this diagnosis in primary health care.

## METHODS

This systematic review was conducted to determine the diagnostic strategies used in primary health care to diagnose dementia and cognitive dysfunction in low-, middle-, and high-income countries. Thus, based on this research question, studies from the past five years were searched on SCOPUS, PubMed, EMBASE, LILACS, SCIELO, and Web of Science. The search occurred in October, 2018, and the key-words used in this study were obtained both from DeCS (Descritores em Ciências da Saúde) and MeSH (Medical Subject Headlines). Country-income classification was based on data from the World Bank website (http://www.worldbank.org/) and adapted to comprise three categories as proposed by the International Association for Media and Communication Research (https://iamcr.org/income). The descriptors were: “dementia”, “cognitive dysfunction”, “diagnosis”, “primary health care”, and “mass screening” - and their correlates in Portuguese and Spanish.

The Boolean operator “AND” was used as a search strategy to combine the descriptors considering all the possibilities. The combinations, in English, were: “Diagnosis AND Dementia AND Primary Health Care”; “Diagnosis AND Cognitive Dysfunction AND Primary Health Care”; “Dementia AND Primary Health Care AND Mass Screening”; “Cognitive Dysfunction AND Primary Health Care AND Mass Screening”. The same combinations were employed in both Portuguese and Spanish.

To make the search more precise, the following filters were applied: papers written in English, Spanish, or Portuguese; publication date from 2014 up to the time of the search (October, 2018). The limit of five years was established due to the improvement and recent discoveries that have been made in the field of dementia screening and diagnosis. On SCOPUS and EMBASE, the required document type was article, and the search was conducted by article, title, and key-word. On PubMed and Scielo, the search was conducted for all fields. On LILACS, the search was by words. Finally, on Web of Science, articles were searched by topic.

After the search, a data base was created by two different researchers. The purpose was to minimize errors and bias. After both data bases were complete, another researcher compared them to ensure they were the same. The selection process was based on the Preferred Reporting Items for Systematic Review and Meta-Analysis (PRISMA) protocol. PRISMA was chosen to accomplish careful planning and organizing data to ensure a review with rigor and quality.^12^ Also, an adapted version of an instrument proposed by URSI (2005) was used for data extraction and analysis. From the findings obtained by the above mentioned instrument, results were organized in a table to facilitate data descriptive synthesis.

For this review, the inclusion criteria considered studies from the previous five years; published in English, Portuguese, or Spanish; conducted in primary health care services; whose participants were aged 60 or older; availability (possible to access); and studies whose topic addressed either diagnosis/screening of dementia or cognitive dysfunction. On the other hand, exclusion criteria were: duplicated articles; drug trials, literature reviews, letters to the editor, editorial, recommendations, monographies, dissertations, and thesis; as well as for articles whose topic did not involve the diagnosis of dementia or cognitive dysfunction.

Because this study was based on published articles, submission to the Research Ethics Committee was not required, according to Brazilian National Health Council’s resolution (nº510/2016).^13^

## RESULTS

**Figure 1:**
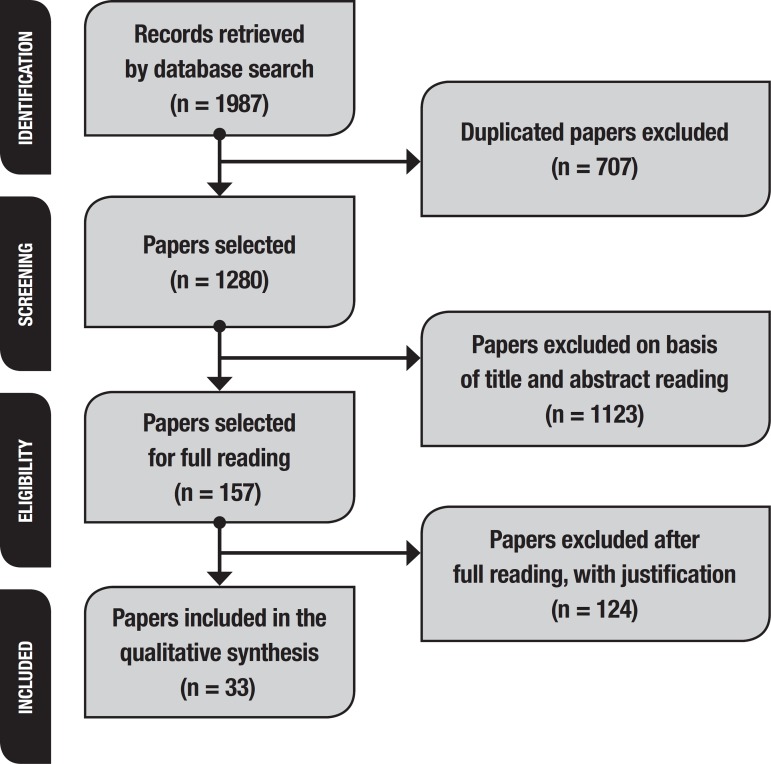
Summary of Paper Selection Process, PRISMA, São Carlos, São Paulo, Brazil, 2019.

The search of the databases retrieved a total of 1987 articles. As mentioned above, PRISMA was the tool used for the selection process. Of the initial total found, 707 papers were excluded because they were duplicated (inter or intra-database). After this exclusion, 1280 remained for title and abstract reading. In this phase, a further 1123 papers were excluded, and 157 articles were selected for full reading. Of this total, 124 documents did not meet the inclusion criteria and therefore 33 studies were included in this systematic review. Results from PRISMA can be seen in Figure 1.

This study’s initial question was “what are the diagnostic strategies to diagnose dementia and cognitive dysfunction in primary health care in low-, middle-, and high-income countries?”. Results showed that more than 90% (n = 30) of the articles were from high-income countries, while 3 papers were from middle-income countries. Unfortunately, no articles from low-income countries were found.

Regarding participants’ demographic characteristics, most of the articles (n = 21) had a predominance of female participants. Age was also analyzed. In general, participant age ranged from 70 to 80 years. Studies conducted in middle-income countries considered older adults as participants aged 60 or older. From the pool of selected studies, 75.8% had between 101 and 1,000 participants; 18.2% had between 1,001 and 10,000; finally, the percentage of studies whose number of participants was more than 10,001 was 6.0%. It was noted that studies often failed to describe participants’ ethnicity. Of the studies that provided this information, Hispanic, African American, Chinese, and White ethnicities were reported.

Because the topic of interest in this study was dementia/cognitive dysfunction diagnosis in primary health care, the type of diagnosis was a variable of interest. After the analysis, three diagnosis categories were established: dementia only (n = 10), MCI only (n = 8), and dementia and MCI (n = 15). Regarding the diagnostic criteria, all of the papers (n = 33) reported clinical diagnosis, conducted either by a general practitioner or a multidisciplinary group, where 13 articles used DSM-IV as the reference criteria. Three studies had different criteria sources for dementia and MCI. In these studies, dementia diagnosis was based on DSM-IV, whereas MCI was based on recommendations of experts (e.g. Petersen et al., and Winblad et al.). For biomarkers, three studies used blood measurements and one study used neuroimaging. Of the total, 9 articles mentioned only neuropsychological testing as a criterion for screening or diagnosing dementia and MCI. Interestingly, all the studies conducted in middle-income countries had this characteristic.

**Graph 1:**
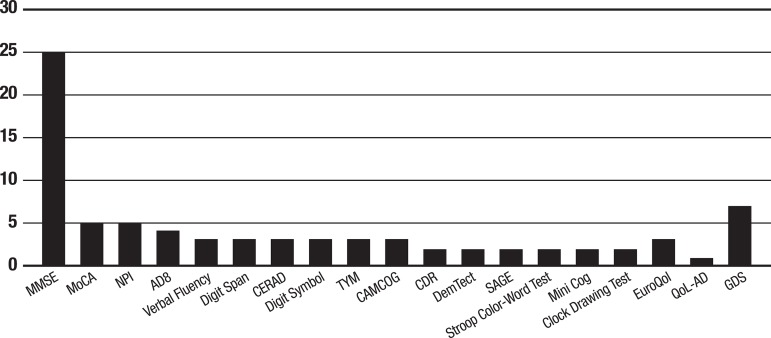
Measurement Instruments used in the studies, São Carlos, São Paulo, Brazil, 2019.

This review also investigated the instruments used for assessing patients’ neuropsychological status and others aspects (e.g. functioning, quality of life, and comorbidities). Graph 1 shows a schematic representation for the most used instruments by the studies. Cognitive instruments were cited in 31 out of the 33 articles; however, only 14 papers mentioned other types of evaluation (non-cognitive). Most of these evaluations reported measurements for quality of life, activities of daily living, and health status. Regarding cognitive assessment, 25 studies used the MMSE as one of the instruments for measuring cognition, and 23 used the MMSE together with another type of cognitive measure. MMSE was the most used instrument. In addition, 5 papers used the MoCA and NPI; 4 papers used the AD8; 3 papers used verbal fluency, digit span, CERAD, digit symbol, test your memory, and CAMCOG tests; 2 papers used the CDR, DemTect, Stroop color-word test, Mini-Cog and the Clock Drawing Test. Quality of life was assessed by the EuroQol in 3 studies and by the QoL-AD in one study. Depressive symptoms were evaluated by the GDS in 7 studies.

The number of diagnosed older adults was also an outcome of interest. Only one study did not provide this information. In total, ten studies investigated the diagnosis of dementia. One did not provide information about the number of diagnosed participants. In three studies, all participants were diagnosed as having dementia. In the other six articles, dementia diagnosis rate ranged from 3.2% to 55%. Furthermore, MCI diagnosis ranged from 15.2% to 55.8% among those studies which investigated this condition only (n = 8). In studies that investigated both dementia and MCI, the number diagnosed with MCI was higher than the number diagnosed with dementia. Appendix 1 shows the information obtained from the analysis of the articles selected for this systematic review.

Also, some articles evaluated the number of patients that did not test positive on screened or diagnosis for dementia/MCI in primary health care. One study suggested that the elderly were considerably underdiagnosed in primary health care. Similarly, another article stated that the rate for underdiagnosed older adults was around 60%.

**Figure 2:**
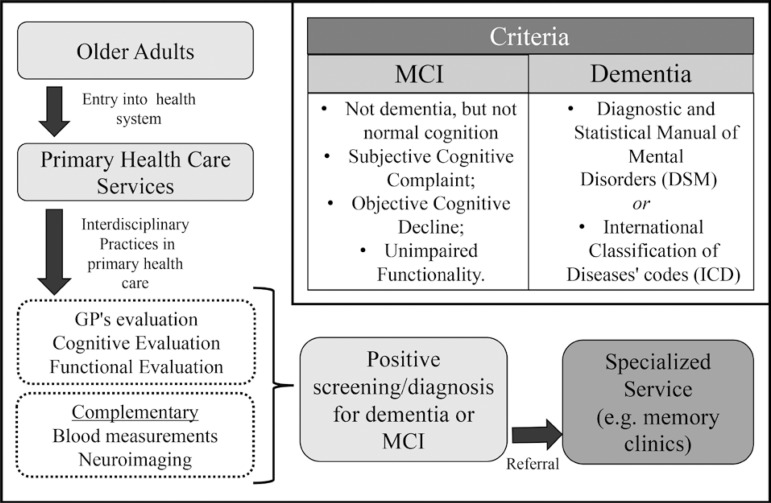
Practice for the diagnosis of dementia and cognitive impairment in high-income countries´primary health care.

The qualitative analysis revealed that high-income countries usually use a manual (e.g. DSM), in addition to cognitive and functional instruments, as well as general practitioners’ evaluation, to establish a diagnosis of dementia in primary health care, for further referral to specialized care. On the other hand, middle-income countries seemed to use only neuropsychological instruments (e.g. MMSE). Figure 2 shows a scheme of diagnostic criteria used in high-income countries that should be helpful for general practitioners when evaluating or screening older adults for MCI or dementia in primary health care.

## DISCUSSION

In this systematic review, studies about the diagnosis of dementia and MCI in primary health care were mostly from high-income countries. In addition, no studies in low-income countries were found. Although dementia is recognized as a global public health issue, poor countries face more difficulties diagnosing and treating this syndrome.^14^ This could be explained by the fact that in low-income countries, health facilities are more often located in big cities, whereas there are few professionals practicing both in the countryside and rural areas.^15^ Also, lack of economic and medical resources, poor training, and lack of expertise in mental health are the main factors contributing to poor care for the elderly, especially those with dementia.^14^^,^ 16 Another possible explanation for the absence of studies in low-income countries may be related to the limited access to health services, as well as the limited creation and implementation of public health policies that contribute toward both patient diagnosis and treatment.^14^^,^ 15 ^,^ 17

Regarding demographic information, the mean age observed in this review (70-80 years) follows the pattern in the literature, which shows that the prevalence of dementia is higher for the oldest elderly.^18^ Research has suggested age as an important risk factor for the development of dementia because, in most cases, it affects individuals aged 65 or older.^19^^,^ 20

It was also observed that high-income countries define older adults as those who are 65 years old or over. This is mainly defined by the increase in life expectancy, as well as the elderly’s better socioeconomic and health conditions.^18^ Because biological age is not always enough to define old age, the World Health Organization has established the age of 60 years old or over for low- and middle-income countries and 65 or over for high-income countries.^15^^,^ 21

In this review, studies reported greater MCI than dementia diagnosis. Although much progress needs to be made in order to solve underdiagnosis problems, research has suggested that MCI is indeed more prevalent than dementia in older adults.^18^^,^ 22 ^,^ 23 Regarding diagnostic criteria, most of the studies used DSM-IV as a guideline. It is important to mention that there is a new edition, DSM-V, but the studies reviewed probably used the previous version because the fourth edition was the only version available at the time the studies were conducted. Also, this manual was shown to be used in high-income countries. Middle-income countries used cognitive evaluation instruments. According to Parra et al.,^15^ middle- and low-income countries have shown a tendency to accept international recommendations for dementia; however, the authors suggested that lack of financial support, resources, trained professionals, and the inexistence of primary health care programs make it difficult to follow these standards.

As the strategy for screening older adults for cognitive decline, most of the articles in this review cited GP evaluation. Only a few studies mentioned a multi-professional group. However, different professionals can contribute toward identification of possible cases of MCI and dementia.^24^^,^ 25 Middle-income countries, such as China, have been investing in the use of screening instruments for trained nurses, who are intended to be part of a multi-professional dementia identification network.^14^^,^ 25 It is also noteworthy that a multi-professional approach with the elderly is recommended because this is desirable to achieve effective and comprehensive health care.^26^ In this context, professionals such as gerontologists, nurses, physical therapists, geriatricians, neurologists, occupational therapists, and psychologists are key elements for dementia screening, diagnosis, and management.

Another interesting aspect observed in this study was the different methods for dementia and MCI identification and confirmation. High-income countries had a uniform standard for diagnosis in primary health care. Our results suggest that these countries, in addition to a manual recommendation (e.g. DSM), also employ complementary tests, such as neuroimaging and blood tests. Research has shown that blood tests, neuropsychological evaluation, and patient health history,^27^ as well as neuroimaging,^28^^,^ 29 are relevant for early identification and differential diagnosis. On the other hand, in this review, studies from middle-income countries only cited the use of neuropsychological evaluation. According to Ferri et al.,^14^ this might be explained by the lack of structure and financial resources for primary health care settings in low- and middle-income countries.

Of the neuropsychological tests mentioned in the articles analyzed, MMSE was the most used. It is also the most commonly used test in screening strategies around the world due to its wide acceptance by the scientific and clinical community, and also because of its practicality and breadth of evaluation.^30^ In addition, MMSE advantages include fast administration and availability in various languages.^31^

As mentioned previously, MCI diagnosis was more common than dementia diagnosis. Although the number of diagnosed patients is substantially larger than the prevalence suggested in the literature, it is relevant to observe that some of the studies suggested the existence of undiagnosed older adults in primary health care. For instance, Zaganas et al.^32^ stated in their study that 60% of the older adults remained without a dementia/MCI diagnosis in primary health care until further in-depth neuropsychiatric evaluation. Similarly, Parmar et al.^33^ evaluated medical records from the Canadian primary health care system and found no cases of MCI diagnosis. The authors also mentioned that 41% of dementia cases were not identified in primary health care.^33^ To sum up, Thyrian et al. concluded in their study, that elderly from primary health care are frequently underdiagnosed for dementia and MCI. Thus, there is still much to be done in order minimize the number of undiagnosed people in primary health care.

One limitation of this study was the fact that the study design did not include the number of diagnoses missed in primary health care, in other words, the number of underdiagnosed patients.

In conclusion, this systematic review aimed to describe how low-, middle-, and high-income countries establish diagnoses for dementia and cognitive dysfunction in primary health care. Most of the articles included in this study were from high-income countries, and no articles were published in low-income countries. In high-income countries, diagnosis or screening for dementia and cognitive dysfunction is usually conducted by general practitioners, who used well-established diagnostic criteria and instruments for assessments (cognitive and functional). In addition, some GPs used complementary evaluations, such as blood tests and neuroimaging. On the other hand, studies published in middle-income countries described only the cognitive assessment process. The diagnosis rate of patients was 3.2-55% for MCI and 15.2%-55.8% for dementia.

Studies focusing on low- and middle-income countries should be conducted. It is important to mention that, considering the demographic profile of these countries, the population tends to be aging and dementia cases may increase considerably. Public policies and investment should be made to prepare primary health care professionals for screening and diagnosing dementia. This would improve both the health system and the flow of patients between the different levels of health care.

## APPENDIX 1

**Table:** Main characteristics of the studies selected for analysis, São Carlos, São Paulo, Brazil, 2019.

First author Year, and place	Demographics (n/mean/age/gender)	Diagnosis type	Diagnostic criteria	Percentage of positive screened/diagnosed patients	Main findings
Garcia-Ptacek^29^ 2017, Sweden	3,891 81.1 (±6.6) 63.9% Female	Dementia	GP’s evaluation; ICD-10; neuroimaging; blood testing	100%	CDT and neuroimaging are used in most of GP’s dementia diagnosis in primary health care
Grober^34^ 2016, USA	257 75.8 69.7% Female	Dementia	DSM-IV; interview with family members or friends	25.7%	Screening based on informants to reduce false- positive rates
Noda^28^ 2018, Japan	623 86.9 54.2% Female	Dementia	GP’s evaluation; DSM-IV	27.4%	DSM Score > I or > II reduces errors for dementia identification in primary health care
Tierney^35^ 2014, Canada	263 77.6 (±6.9) 58.55% Male	MCI	GP’s evaluation; MMSE < 26	28.5%	MMSE would improve GP’s capacity to detect MCI in primary health care
Wilcock^27^ 2016, England	136 79.5 64% Female	Dementia MCI	Blood testing, cognitive evaluation	100%	An update of diagnosis records for comprehensive care is needed
Chan^36^ 2016, Singapore	309 71.7 (+-8.2) 50.2% Female	Dementia MCI	DSM-IV	21.3%	Combinations of AD8 and NINDS provided a sensitivity of 73.3% and specificity of 96.9% for dementia and MCI diagnosis, respectively
Eichler^37^ 2014, Germany	243 79.61 (±5.44) 61 % Female	Dementia MCI	DemTect < 9; medical records	Dementia - 40% MCI-58%	Diagnosis rates for dementia in Germany are consistent with international literature
Eichler^38^ 2015, Germany	243 >70 60.9% Female	Dementia MCI	MMSE < 23; DemTect < 9	Dementia - 49%	The diagnosis rate of dementia increased 40%
Arabi^39^ 2016, Malaysia	200 68.5 (±6.28) 52% Female	Dementia MCI	EDQ < 5; MMSE < 21	EDQ-40% MMSE-20%	Validated questionnaire
Shaik^40^ 2015, Singapore	309 71.8 (±8.2) 54.8% Female	MCI	At least one impaired cognitive domain on objective cognitive evaluation	54.8%	Risk factors identified were: age, female gender, hypertension, diabetes, hyperlipidemia and smoking
Booker^41^ 2016, Germany	11,956 80.4 61 % Female	Dementia	Medical Database analysis	100%	The risk factors identified were: diabetes, hypertension, obesity, hyperlipidemia, vascular diseases
Rosenbloom^42^ 2018, USA	87 77.2 (±6.2) 59.8% Female	Dementia MCI	Mini-Cog < 4/5	27.3% among screened positive on Mini-Cog	Twice the percentage previously identified with cognitive impairment
Lee^43^ 2017, Singapore	140 72.15 (±8.42) 68% Male	MCI	MMSE; MoCA	23.5%	Just a small fraction of those considered high risk for developing dementia made use of health services
Corcoles^10^ 2017, Spain	104 77.8 (±6.74) 68.3% Female	MCI	MMSE	55.8%	91.4% of cases with alteration on MMSE had no history of Cognitive Impairment
Holsinger^44^ 2015, USA	186 74.5 (±6.5) 96.2% Male	DementiaCIND	Medical evaluation	Dementia -12% CIND-31%	20% returned to normal cognition, 67% remained impaired, and 12% developed dementia
de Oliveira^45^ 2016, Brazil	102 76.81 (±7.03) 83% Female	Dementia	DSM-IV; medical records; MMSE; CASI-S	46%	Validation of CASI-S with a 93% sensitivity and 81% specificity
Zaganas^32^ 2019, Greece	3,140 73.7 (±7.8) Gender: 56.8% Female	Dementia MCI	DSM-IV	Dementia-10.8% MCI-32.4%	Dementia prevalence was 4%; in primary care 60% remain undiagnosed until detailed neuropsychiatric evaluation
Pujades-Rodrigues^46^ 2018, UK	47,386	Dementia	Medical records	55%	47,386 with dementia, 12,633 Alzheimer Disease, 9,540 vascular disease and 1539 with other less common causes
Malmstron^47^ 2015, USA	533 65-92 100% Male	Dementia MCI	DSMIV	Dementia -12% MCI-26%	RCS sensitivity 89% and specificity 87% for detecting Dementia, compared to 94% and 70% for MCI
Stein^48^ 2015, Germany	3,327 81.14 65.3% Female	Dementia	GP’s and multidisciplinary group’s evaluation; DSM-IV; SIDAM	Follow-up I - 3.2% Follow-up II - 4.62%	MMSE was more accurate than MMSE for diagnosis
Yang^25^ 2015, China	249 67.6 61.8% Female	MCI	MMSE	Impaired cognition -12.9% MCI-41%	Simple instruments, such as MMSE and MoCA used for screening the elderly in primary health care
Shaik^49^ 2016, Singapore	168 80.7 56% Female	Dementia MCI	Nurses’ screening; AD8; Specialist’s evaluation	Screened positive -13.7%	98.8% of nurses considered AD8 easy to use. 78.3% of GPs considered AD8 useful
Thyrian^50^ 2016, Germany	516 80 59.5% Female	Dementia MCI	GP’s evaluation; ICD-10	MCI-90.8% Dementia-99.8%	Older adults from primary health care are considerably underdiagnosed
Koekkoek^51^ 2015, Netherlands	513 >70	MCI	GP’s evaluation; DSM-IV (Dementia); Winblad et al. (MCI)	15.2%	This study protocol describes all the procedures for the Cog-ld study
Chan^52^ 2016, Singapore	309 71.7 (±8.2) 60.5% Female	Dementia	DSM-IV; CDR	36.5%	For participant age, AD8 was better than MMSE and as good as MoCA
Koekkoek^53^ 2016, Netherlands	228 76.8 60% Male	MCI	DSM-IV (Dementia); Winblad et al. (MCI)	19.3%	TYM’s negative predictive value (NPV) was 81% and SAGE’s was 85%. GP’s evaluations had a similar NPV, however, the positive predictive value was higher
Dungen^54^ 2015, Netherlands	647 79.8 (±7.1) 39.6% Male	Dementia MCI	DSM-IV (Dementia); Petersen at al. (MCI)	Dementia -14% MCI-31.5%	The authors did not find statistical relevance in the number of diagnoses between the groups before or after intervention
Groeneveld^55^ 2018, Netherlands	120 77.0 (±4.5) 60% Male	Dementia MCI	DSM-IV (Dementia). MCI: not dementia, but not normal cognition; cognitive complaints; objective impairment in one or more cognitive domain; no functional impairment	Dementia-2.5% MCI-30%	The authors suggested that patients with type 2 diabetes should be screened for MCI and dementia.
Campbell^56^ 2018, USA	350 71.2 (±5.1) 79.1 % Female	Dementia MCI	Multidisciplinary group evaluation	Dementia - 2% MCI-94.8%	The use of anticholinergic drugs increased the likelihood of conversion from normal to MCI. On the other hand, reversion from MCI to normal cognition was not observed.
Jessen^57^ 2014, Germany	2,892 79.7 (±3.58) 64.8% Female	Dementia MCI SMI	CERAD’s verbal memory task (SMI, eMCI, and IMCI); DSM-IV, SIDAM (Dementia)	Ml - 36.6% eMCI - 8.6% MCI-12.3% DA-7.4%	The highest risk for developing dementia was in the late MCI group. In SMI and early MCI groups, those who had concerns about their memory impairment had a similar risk for developing dementia.
Wray^58^ 2014, USA	5,333 80.7 97% Male	Dementia	Medical records	Not mentioned	B0MC+ patients were 5.12 times more likely to receive a dementia diagnosis, when comparing to BOMC- group.
Alonso^59^ 2016, Spain	4,360>65	MCI	Mini-cog screening test, MMSE and Alzheimer’s Questionnaire	18.5%	Cognitive impairment is a common reason for appointments in primary health care.
Brodaty^60^ 2016, Australia	1,717 81.05 (±4.12)	Dementia	Medical records, MMSE	7.3%	GPCOG’s sensitivity was 79% and specificity 92%.

MCI: Mild Cognitive Impairment; CIND-Cognitive impairment not-dementia; SMI -Subjective Memory Impairment; eMCI: Early Mild Cognitive Impairment; lMCI: Late Mild Cognitive Impairment; GP: General Practitioner; ICD: International Statistical Classifi cations of Diseases and Related Health Problems; DSM: Diagnostic and Statistical Manual of Mental Disorders; MMSE- Mini-Mental State Examination; MoCA: Montreal Cognitive Assessment; CASI-S: Cognitive Abilities Screening Instrument-Short Form; EDQ: Early Dementia Questionnaire; SIDAM - Structured Interview for the diagnosis of Dementia of the Alzheimer type; CDR: Clinical Dementia Rating; GPCOG: General Practitioner assessment of Cognition.
